# Whispering Gallery Modes in Standard Optical Fibres for Fibre Profiling Measurements and Sensing of Unlabelled Chemical Species

**DOI:** 10.3390/s100301765

**Published:** 2010-03-03

**Authors:** Anna Boleininger, Thomas Lake, Sophia Hami, Claire Vallance

**Affiliations:** Department of Chemistry, University of Oxford, Chemistry Research Laboratory, 12 Mansfield Rd, Oxford OX1 3TA, UK

**Keywords:** chemical sensor, whispering gallery mode, optical fibre

## Abstract

Whispering gallery mode resonances in liquid droplets and microspheres have attracted considerable attention due to their potential uses in a range of sensing and technological applications. We describe a whispering gallery mode sensor in which standard optical fibre is used as the whispering gallery mode resonator. The sensor is characterised in terms of the response of the whispering gallery mode spectrum to changes in resonator size, refractive index of the surrounding medium, and temperature, and its measurement capabilities are demonstrated through application to high-precision fibre geometry profiling and the detection of unlabelled biochemical species. The prototype sensor is capable of detecting unlabelled biomolecular species in attomole quantities.

## 1. Introduction

Whispering gallery mode (WGM) resonances have been observed in liquid droplets and a variety of man-made resonators, including microspheres, toroidal resonators, and of particular interest for the work presented in this paper, optical fibres. The exquisite sensitivity of such resonances to the shape and size of the resonator and also to the refractive indices of the resonator and the surrounding medium [1] have led to their exploitation for a wide variety of applications. These range from droplet and fibre size measurements [2–5] and various types of strain [6], acceleration [7, 8] and temperature gauges [9–11], to sophisticated optoelectronics components, including tunable filters [12], optical switches [13], and even lasers [14, 15].

Recently, there has been increasing interest in the potential applications of whispering gallery modes in the field of chemical and biochemical sensing. The field has recently been reviewed by Vollmer and Arnold [16]. Sensors may be grouped into two general classes. The first type uses the WGM resonator as a multi-pass monochromatic evanescent-wave excitation source for fluorescently-tagged surface bound molecules, providing a large enhancement in the fluorescence signal over single-pass techniques [17]. Microsphere-based sensors have also been developed that detect adsorbed molecules based on the frequency shift they induce in a single resonant mode [18–20] or through their spectroscopic absorption signatures [21]. Recently, Vahala and coworkers have claimed that single molecule detection sensitivities are possible for unlabelled biomolecules when WGM frequency shifts are monitored in custom-fabricated toroidal resonators [22]. While the claims of single molecule detection have recently been challenged following theoretical work by Arnold *et al.* [24], the exceptional detection sensitivity achievable using WGM techniques is undisputed. The success of WGM approaches arises from a combination of the exceptional sensitivity of the WGM frequencies to refractive index and the fact that the long effective path length of the WGM within the resonator provides multiple opportunities for interaction between the circulating light and the adsorbed species.

Here we describe a whispering gallery mode sensor based on excitation of whispering gallery modes in a standard communications-grade optical fibre. We provide a brief introduction to the physical principles behind whispering gallery mode spectroscopy, before describing our experimental setup and a range of experiments carried out to characterise the technique and explore its potential applications. We demonstrate the sensitivity of the spectrometer to fibre diameter and to surface refractive index, characterise the response to temperature, and present proof-of-concept data on using fibre-based WGM spectroscopy for targeted sensing of an unlabelled protein. To our knowledge, while cavity enhanced absorption spectroscopy has recently been demonstrated using a fibre-based microcavity [23], chemical sensing using WGMs excited in bare optical fibres in combination with targeted adsorption has not been reported previously.

## 2. Theory

WGM frequencies in an optical fibre, or indeed any cylindrical resonator, are reasonably well approximated by matching an integral number of wavelengths to the resonator circumference, *i.e.*,

(1) $$m(\lambda /n)=\pi d\;\;\;\;\;\;\;\;\;\text{or}\;\;\;\;\;\;\;\;\;\;\;\;\nu =\frac{\mathrm{mc}}{\pi \mathrm{dn}}$$

where *m* is an integer (the azimuthal mode number, discussed further below), *λ* is the free space wavelength and *ν* the frequency of the light used to excite the resonator, *n* is the refractive index of the resonator, and *d* is the resonator diameter. This treatment becomes more accurate as *m* increases and the path taken by the light within the resonator becomes more closely approximated by a circle. A key result is that both the WGM frequencies and the frequency spacing between adjacent modes are inversely proportional to the resonator diameter. However, in reality the true path is ‘polygonal’ in nature, such that the path length is shorter, and therefore the resonant frequency higher, than predicted by this simple model.

The true mode frequencies within a cylindrical resonator are eigenvalues of the appropriate wave equation. The solutions to such an equation take the form of Bessel functions (also known as cylindrical harmonics) and the whispering gallery modes are defined by three mode numbers: the radial mode number, *l*; the azimuthal mode number, *m*; and the slab mode number, *p* [25]. A WGM described by mode numbers *m*, *l* and *p* has 2*m* field maxima around the cylinder azimuth (*i.e.*, around the circumference of the cylinder), *l* − 1 nodes along the radial coordinate of the cylinder, and *p* nodes along the cylinder axis. Outside the resonator, the field decays exponentially with a decay constant *d* of approximately [26]

(2) $$d=\frac{\lambda }{2\pi {\left(n_{a}^{2}\;{\text{sin}}^{2}\theta -n_{b}^{2}\right)}^{1/2}}$$

where *λ* is the wavelength, *n_a_* is the refractive index of the resonator, *n_b_* is the refractive index of the surroundings, and *θ* is the angle at which total internal reflection takes place at the internal surface of the resonator. We see that the evanescent field from the resonator decays over a distance scale on the order of a wavelength. This has the important consequence that WGM sensing techniques are sensitive only to chemical species in close proximity to the sensor surface.

The measure of an optical resonator or cavity’s ability to confine light of a given frequency *ν*_0_ is usually expressed in terms of a ‘quality factor’ or ‘Q-factor’, *Q*.

(3) $$Q=2\pi \nu_{0}\frac{P_{\text{stored}}}{P_{\text{lost}}}=\frac{\nu_{0}}{\delta \nu }$$

where *P*_stored_ and *P*_lost_ are the power stored within the cavity and lost from the cavity, respectively, and *δν* is the spectral linewidth of the cavity mode at frequency *ν*_0_. Gorodetsky *et al.* [27] have calculated that the ultimate *Q* of a perfect microsphere resonator is around 10^10^. Experimentally measured *Q* factors are generally much lower than this due to scattering from surface roughness and impurities, absorption by the material, and simultaneous excitation of multiple closely spaced modes. Cylindrical resonators have been characterised with *Q* factors of the order of 10^5^ or higher [28].

The response of the whispering gallery mode spectrum to molecular binding at the resonator surface has been explained in terms of a change in optical path length caused by direct interactions between the resonant photons and the bound molecules. When a molecule binds to the resonator surface, it displaces water and creates an excess dipole moment at the binding point. Arnold *et al*. [19] considered the interaction energy between this excess dipole and the evanescent field of the WGM resonances, and converted the resulting energy loss from the WGM field into a fractional shift in the corresponding resonance frequency. According to this model, the shift *δν* associated with binding of *N* randomly located molecules to the resonator surface is given by

(4) $$\frac{\delta \nu }{\nu }=-\frac{\alpha_{\text{ex}}\sigma_{P}}{2{ɛ}_{0}{ɛ}_{\text{rs}}}\frac{\int {\left|E_{0}(r)\right|}^{2}\mathrm{dA}}{\int {\left|E_{0}(r)\right|}^{2}\mathrm{dV}}$$

where *α*_ex_ is the excess polarisability, *σ*_p_ is the surface density of adsorbed molecules, **E**_0_(**r**) is the electric field at position **r**, ɛ_0_ is the permittivity of free space, and ɛ_rs_ is the relative permittivity of the resonator. The integrals are over the surface area and volume of the whispering gallery mode, with the volume integral yielding the total energy of the mode within the resonator. Of key importance is that the fractional shift on binding depends on both the fraction of the mode energy found at the positions of the bound molecules, and on the excess polarisability of the molecules. For proteins, *α*_ex_ is approximately proportional to the molecular mass, such that larger shifts are predicted for heavier molecules.

White and Fan [29] have generalised this result. For a sensor with a sensitivity *S* = *δλ/δn* to changes in the refractive index of the bulk, where *δλ* is the observed wavelength shift accompanying a change *δn* in refractive index, the fractional wavelength shift on molecular binding to the sensor surface is

(5) $$\frac{\delta \lambda }{\lambda }=\sigma_{P}\alpha_{\text{ex}}\frac{2\pi {\left(n_{m}^{2}-n_{s}^{2}\right)}^{1/2}}{{ɛ}_{0}\lambda^{2}}\frac{n_{m}}{n_{s}^{2}}S$$

Here, *n*_m_ is the refractive index of the sensor material and *n*_s_ is the refractive index of the solvent. This expression allows the surface density of detected molecules to be determined from the measured wavelength shift.

The detection limit is highly sensitive to the *Q* factor of the cavity, since a higher *Q* leads to sharper cavity mode spectra and detection of smaller shifts in resonance frequency. A consequence of this is that the detection limit of a fibre-based whispering gallery mode sensor is likely to be somewhat lower than for microsphere-based sensors, due to the lower Q factor of cylindrical relative to spherical cavities. However, there are a number of potential advantages that make fibre-based sensors worth exploring. Some of these have been pointed out previously by Farca *et al.* [23]. Optical fibres are highly uniform in diameter, allowing large numbers of identical resonators to be fabricated and providing a high degree of repeatability. An additional advantage is the straightforward optical setup for fibre-based experiments. Fibres are easily mounted, and alignment for optimal coupling of the excitation light into the fibre WGMs depends on only one angular degree of freedom, as opposed to two for experiments involving microspheres.

## 3. Experimental

**Figure 1.**
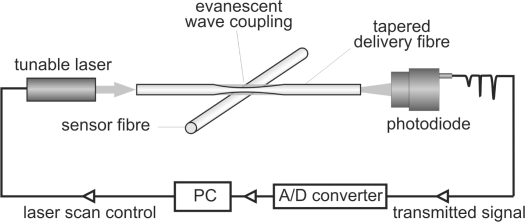
Schematic of the experimental setup for optical fibre whispering gallery mode spectroscopy. Light from a tunable laser is coupled into a tapered optical ‘delivery’ fibre in contact with a ‘sensor’ fibre. As the laser wavelength is scanned, light coupled into whispering gallery mode resonances in the sensor fibre leads to periodic dips in the optical signal transmitted through the delivery fibre.

The basic experimental setup for recording the WGM spectrum of an optical fibre is shown schematically in Figure 1. The fibre to be probed is labelled in the figure as the ‘sensor fibre’. Except where otherwise stated, the sensor fibre was a standard 125 *μ*m diameter single mode optical fibre with its plastic buffer coating removed. Buffer removal was achieved either using a standard fibre stripper or by placing the fibre in the flame of a butane torch. The small core diameter of a single mode fibre (∼8 *μ*m), together with the fact that the WGMs excited are predominantly confined near to the surface, means that the sensor fibre essentially acts as a uniform cylindrical dielectric with the refractive index characteristics of the fibre cladding. Light is coupled into WGMs of the sensor fibre via the evanescent field generated in a tapered region of a second fibre, labelled as the ‘delivery fibre’ in Figure 1. Tapered regions in the delivery fibre are fabricated by the ‘heat and pull’ method using a home-built, computer controlled tapering rig. The fibre to be tapered is clamped between a pair of programmable linear translators, providing fine control over the rate at which the fibre is pulled and the final extension. Heat is provided by a small butane torch mounted on a third linear translator positioned at the centre of the two fibre clamps. After ignition, the flame is moved into contact with the fibre, the fibre is pulled to the desired length, and the flame is moved away and extinguished. An extension of 15–20 mm is generally sufficient to achieve a sub-micron taper in a 125 *μ*m fibre. Transmission losses through the fibre on tapering varied depending on the exact tapering conditions and final taper diameter, but were generally of the order of a few tens of percent.

The light source used to excite whispering gallery modes in the sensor fibre is a 20 mW fibre-coupled Santur TL2020-C DFB communications laser, tuneable over the range from 191.7 to 196.1 THz in 0.05 THz steps (1564 – 1529 nm in ∼0.4 nm steps), and the detector is a DET10C high speed InGaAs photodiode sensor (Thorlabs). Light from the laser source is coupled into the input end of the delivery fibre, and the signal at the output end is detected by the photodiode. The measured signal as a function of wavelength is recorded on a PC via a USB analogue to digital converter (LabJack U12). In the absence of a sensor fibre, the measured signal is uniform with wavelength and simply reflects the emission spectrum of the laser, modified somewhat by the transmission characteristics of the delivery fibre. To measure the WGM spectrum of a sensor fibre, the fibre is crossed at right angles with the tapered region of the delivery fibre and the two fibres are brought into contact. Each time the laser wavelength comes into resonance with a WGM, light is coupled efficiently from the delivery fibre into the sensor fibre. This reduces the amount of light transmitted through the delivery fibre, resulting in a dip in the measured signal intensity at each resonant wavelength.

The detailed mode structure of the WGM spectrum depends on the particular combination of delivery and sensor fibre used, since small variations in taper geometry can have quite marked effects on the relative coupling efficiencies into different modes. This may be seen in the data presented in Figures 3, 6 and 7, for which different taper-sensor pairs were used in the various experiments described. However, for a given taper-sensor pair the two fibres may be repeatedly separated and brought into contact with high reproducibility of the resulting WGM spectra. Damage to the fibre surfaces on contact therefore does not appear to be a matter for concern, and more complicated experimental setups in which the fibres are held in close proximity but not in contact are not necessary.

Details of individual experiments will be described along with the results in Section 4.

### 3.1. FDTD Simulations

Efficient coupling between the evanescent field generated at the surface of the tapered delivery fibre and a whispering gallery mode in the sensor fibre requires optimum overlap between the two mode fields. This is strongly dependent on the diameters of the two fibres, and in particular on the diameter of the tapered delivery fibre. To determine the optimum taper diameter, a two-dimensional model of the crossing region was simulated using a commercial finite difference time domain (FDTD) software package [30]. Two-dimensional simulations clearly cannot model coupling into axial modes of the fibre (*i.e.,* the simulations are limited to modelling *p* = 0 modes), so the simulated spectra are somewhat simpler in form than many of our measured spectra. However, with the exception of experiments carried out specifically to investigate coupling into axial modes, described in the next section and illustrated in Figure 3, these are generally a fairly small contribution to our signal.

**Figure 2.**
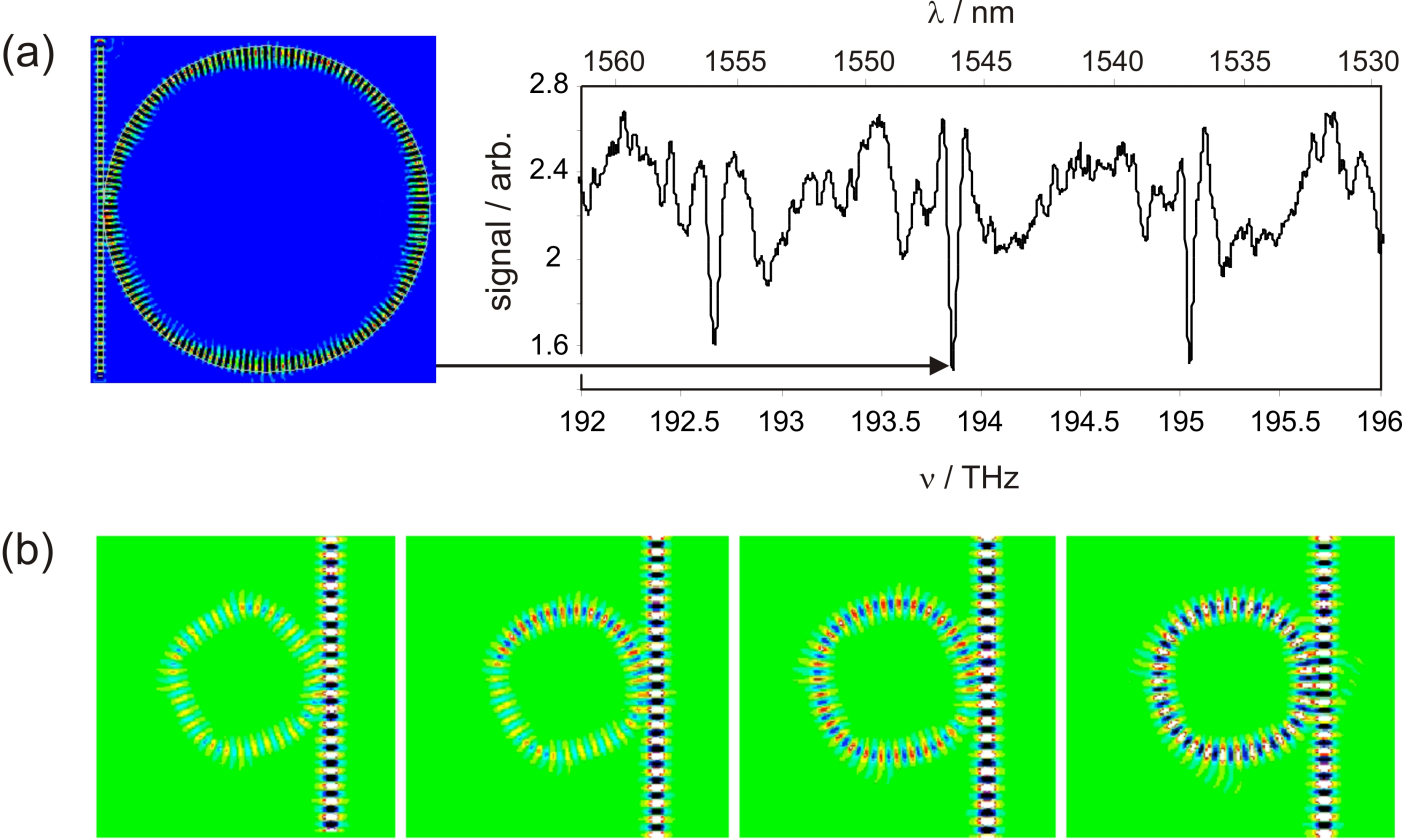
2D finite difference time domain (FDTD) simulations of WGMs in optical fibres. Panel (a) shows part of the simulated spectrum for a 50 micron sensor fibre, with the electromagnetic field for a selected mode (*m* = 146, *l* = 1) shown inset; panel (b) illustrates the build up of the electromagnetic field in the *m* = 31, *l* = 1 WGM of a 10 micron fibre through constructive interference over time.

The frequency resolution of the simulations is controlled via a Fourier transform relationship by the total time period simulated, and was chosen to match the frequency resolution of our experiments. The computational requirements of the FDTD code limited simulations to fairly small resonator diameters, even for two-dimensional simulations. However, while we were not able to carry out simulations matching our experimental frequency resolution for a 125 *μ*m fibre, there was considerable overlap between the range of fibre sizes for which we could perform simulations and the range of sizes used experimentally in the fibre profiling experiments described below. Simulations run with a range of resonator diameters (with a background refractive index corresponding to that of air) indicate that coupling is inefficient until the taper diameter falls to around one micron or less. This is in line with our experimental observations and with the taper dimensions used by other authors to excite WGMs in cylindrical cavities [31]. While other authors have shown that larger diameter tapers may be used to couple light into whispering gallery modes of microspheres [32, 33], in our experiments with bare fibre resonators we were consistently unable to observe spectra using taper diameters larger than a micron. Figure 2(a) shows a simulated WGM spectrum for a 50 *μ*m diameter sensor fibre excited by the evanescent field from a 1 *μ*m diameter taper. The simulation software also allows the electric field distribution within the two fibres to be plotted, which provides a useful visualisation of the mode field overlap between the delivery and sensor fibres. Build-up of the electric field inside the resonator with time during excitation at a frequency corresponding to a WGM is shown in Figure 2(b).

The simulations also allow us to examine the coupling between the delivery and sensor fibres when the fibres are in contact and when they are separated. Since the evanescent field from the delivery taper decays exponentially away from the surface, coupling is unsurprisingly stronger at very small separations and when the fibres are in physical contact. Another important observation is that there is very little perturbation of the electric field of the WGM inside the sensor fibre as the delivery fibre is brought into close proximity or even into contact.

Currently, the resolution of the instrument is limited by the frequency resolution of the communications laser used to make the measurements. In the next phase of the project, the tuneable laser/photodiode setup will be replaced with a broadband supercontinuum source and high resolution spectrometer. This will not only improve the instrument resolution, but will also greatly increase the speed of data acquisition, since wavelength scans will no longer be necessary. However, here we will demonstrate that even with the relatively low resolution currently attainable, the instrument provides a powerful tool for the characterisation of optical fibres, and we will also provide what we believe is the first proof of concept for targeted chemical sensing using whispering gallery modes in optical fibres.

## 4. Results and Discussion

### 4.1. WGM Spectra of Bare Fibres in Air

**Figure 3.**
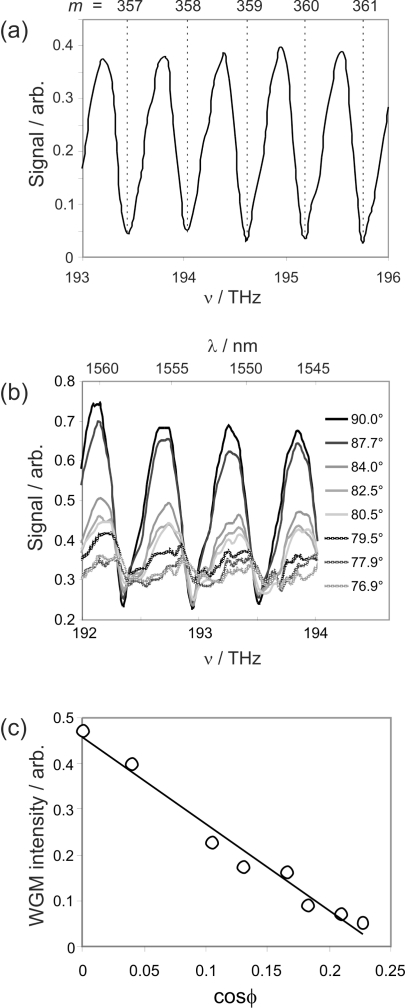
(a) Experimentally measured WGM spectrum for a 125 *μ*m diameter bare fibre; (b) spectra recorded at a range of contact angles between the delivery and resonator fibres; (c) cos *ϕ* dependence of the light intensity coupled into whispering gallery modes on the crossing angle between the delivery and resonator fibres.

Figure 3(a) shows a WGM spectrum recorded for a standard 125 *μ*m diameter single mode fibre in air. The spectrum is seen to consist of a series of equally spaced dips, corresponding to WGMs of successively increasing azimuthal mode number *m* (mode numbers are labelled on the top axis). A lower limit for the Q factor of our fibre resonators may be determined from the linewidth of the measured whispering gallery modes to be around 1.5 × 10^3^. Given the limited resolution of the laser currently being used, and our present inability to resolve individual sub-modes as a result (as discussed in the following paragraph), the true Q factor is almost certainly considerably higher than this. An indication of the potentially realisable *Q* factors in our configuration may be obtained from the FDTD simulations. The simulations described above were carried out at a resolution approximately matching that of the experiments. However, simulations carried out at higher resolution predict a *Q* factor of 1.73 × 10^5^ for a 50 *μ*m resonator at an excitation wavelength of 1558 nm. Since 2D FDTD simulations are unable to account for longitudinal dispersion of the WGM along the fibre axis, it is likely that the true value lies somewhere in between our experimentally measured value and that predicted by the simulations. For comparison, Q factors reported for WGMs excited in thin-walled capillaries by Zamora *et al.* [31] and White *et al.* [34] range from around 500 up to 6.3 × 10^5^, while planar disk resonators[35–37] have typically been found to have *Q* factors of around 10^4^.

The modes that are most efficiently coupled into using the excitation geometry employed have *l* = 1, *p* = 0 (*i.e.,* no radial or axial nodes), though the visible shoulder to the high frequency side of each peak may almost certainly be attributed to unresolved higher order modes in *l* and *p*. This may be explored further by examining the effect of crossing angle (*i.e.,* the angle of the sensor fibre relative to the delivery fibre at the crossing point) on the spectra. Reasonably accurate values for the coupling angle in our experiments may be determined from video microscope images of the crossed fibres. A number of spectra recorded at different crossing angles by mounting the resonator on a rotating stage are shown in Figure 3(b). The overall transmitted intensity is seen to decrease as the contact angle *ϕ* moves away from 90 degrees, and the peaks broaden.

The coupling efficiency into a given whispering gallery mode depends on the extent of overlap between the evanescent field of the delivery fibre taper and the mode of interest inside the resonator. When considering the *l* = 1, *p* = 0 mode, the overlap is at a maximum when the two fibres are crossed at right angles. The projection of the delivery fibre field onto the WGM field reduces as the angle is moved away from 90 degrees, explaining the observed reduction in signal from this mode as the crossing angle is reduced. At such geometries, overlap with other helical WGMs of the resonator is improved, and we see increased signal from coupling into these modes. This manifests mainly as a broadening in the spectral peaks. However, structure is clearly visible within the broadened signal, corresponding to the various modes excited.

We can consider coupling into the *l* = 1, *p* = 0 mode on a more quantitative level. The electric field of this mode lies in the plane containing the fibre axis, and the projection of the delivery fibre field onto this axis varies as the cosine of the crossing angle *ϕ* between the two fibres. We may therefore expect the intensity of the *l* = 1, *p* = 0 peak (*i.e.,* the depth of the resonance dip in the signal) to vary in proportion to cos *ϕ*. As shown in Figure 3(c), this is indeed found to be the case.

Taking these results into account, care was taken in all subsequent experiments to ensure that the delivery and sensor fibres were crossed at right angles.

**Figure 4.**
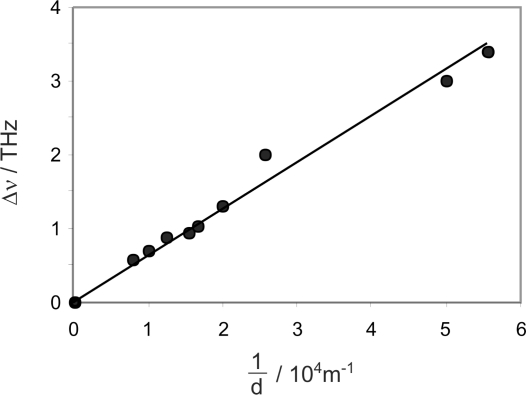
Dependence of the WGM peak separation on sensor fibre diameter. A linear fit to the data forced through the origin has a slope of 6.31 × 10^7^, compared with a value of 6.53 × 10^7^ predicted by Equation 6.

From Equation 1, the free spectral range of the cavity *δν*, *i.e.,* the spacing between adjacent peaks, is given approximately by

(6) $$\Delta \nu =\frac{c}{n\pi d}$$

suggesting that the peak separation should be inversely proportional to the sensor fibre diameter. The refractive index *n* in the above expression could more rigorously be replaced with the ‘effective refractive index’ *n_e_* seen by the propagating WGMs in the resonator. The effective index will have some dependence on the size of the resonator due to the differing evanescent field fractions. However, other authors have shown [38] that for cylindrical resonators in the size range of interest, these variations are fairly small, of the order of one to two percent, so Equation 6 is a good approximation to the true situation. The inverse dependence of the free spectral range on resonator diameter was confirmed by recording spectra for a series of bare fibres tapered to various different diameters, as measured using an optical microscope. The data shown in Figure 4 clearly demonstrate the predicted inverse relationship between Δ*ν* and *d*. The increase in scatter of the data points at large values of 1/*d* is largely due to the difficulty in obtaining an accurate microscopic measurement of the fibre diameter as the diameter reduces. The slope of the plot is 6.31 × 10^7^ m s^−1^, in fairly good accord with the model, which predicts a proportionality constant of $\frac{c}{n\pi }=6.53\times 10^{7}m\;s^{-1}$. The small discrepancy between the two values is due to the effective index effects noted above, combined with the fact that whispering gallery modes are not in reality confined entirely to the surface of the resonator, as is assumed in the simple ‘particle on a ring’ type model, but penetrate varying distances into the interior, thus reducing the effective path length around the resonator circumference relative to that predicted by the model. Since the effective index will be slightly lower than the true refractive index, the two effects cancel to some extent.

### 4.2. Fibre Profiling

**Figure 5.**
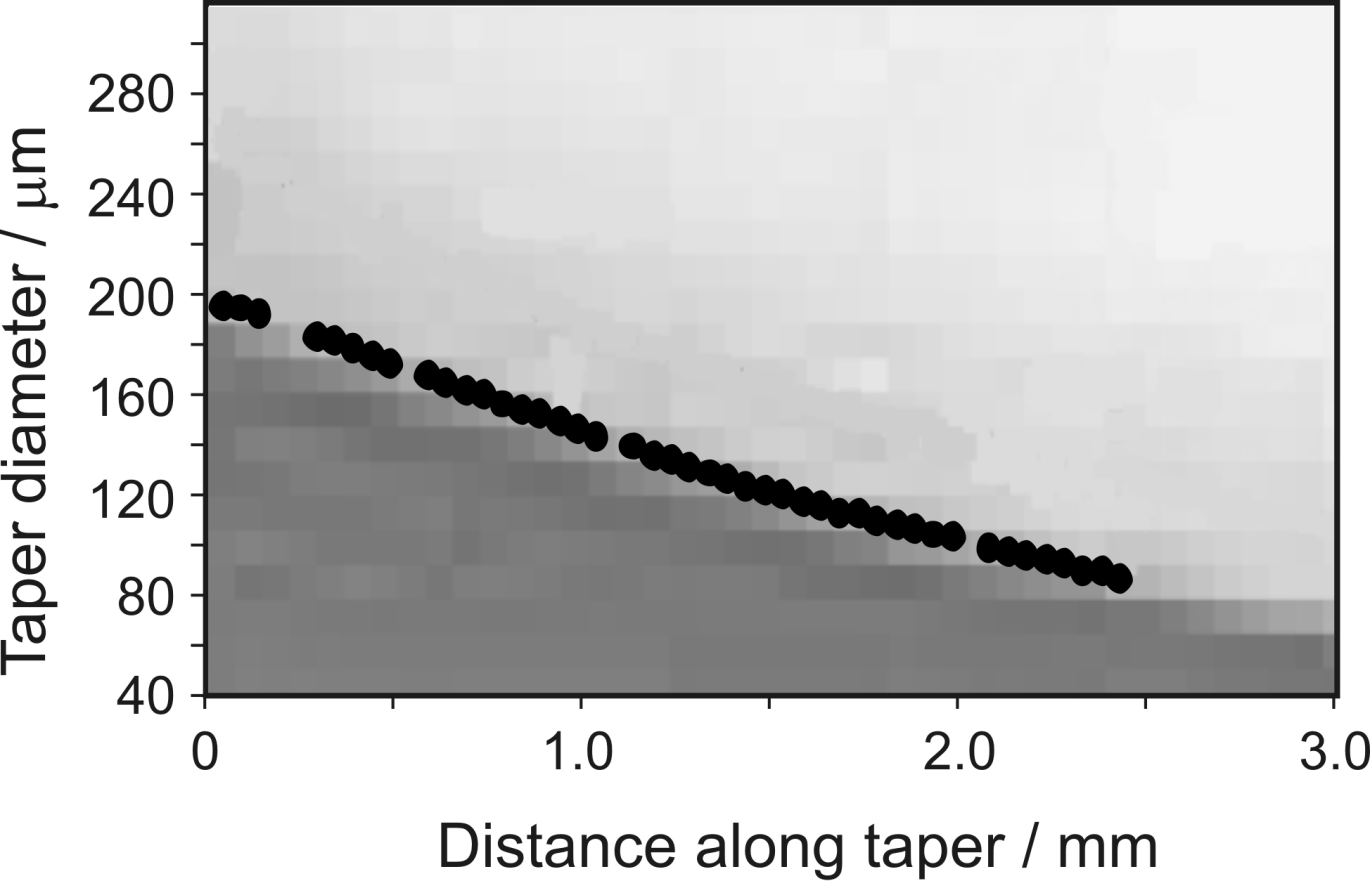
Measurement of the geometrical profile of a tapered optical fibre from the spacing between WGMs measured at a series of positions along the taper. The profile extracted from the spectra is overlaid with a microscopic image of the taper.

The extreme sensitivity of WGM spectra to changes in the resonator shape and size make WGM spectroscopy a promising tool for topological measurements at the sub-wavelength scale. The use of WGMspectroscopy in the characterisation of fibre diameter variations has been demonstrated previously by Birks *et al.* [3] and by Warken and Giessen [4]. In Figure 5, we illustrate the ability of WGM spectroscopy to yield an accurate profile of a tapered optical fibre. To record the profile, spectra were recorded as the delivery fibre was stepped along the tapered fibre under study. The calibration performed above then allows the fibre diameter at a given crossing point to be determined from the frequency spacing of WGMs in the corresponding spectrum. An accurate value for the frequency spacing was obtained from a Fourier analysis of the spectrum.

The results of this approach are validated by comparing the taper geometry determined from these spectroscopic measurements with a microscopic image of the taper; the two sets of data are overlaid in Figure 5, and show excellent agreement. A determination of the standard deviation of our measurements about a ‘best fit’ line through the measured profile yields a value of around 300 nm, indicating that even with the modest frequency resolution attainable from our tuneable laser source, we are able to resolve sub-micron changes in the fibre diameter. As demonstrated by Birks *et al.* [3], improving our laser resolution will allow measurements to be made with even higher precision.

### 4.3. Targeted Chemical Sensing

**Figure 6.**
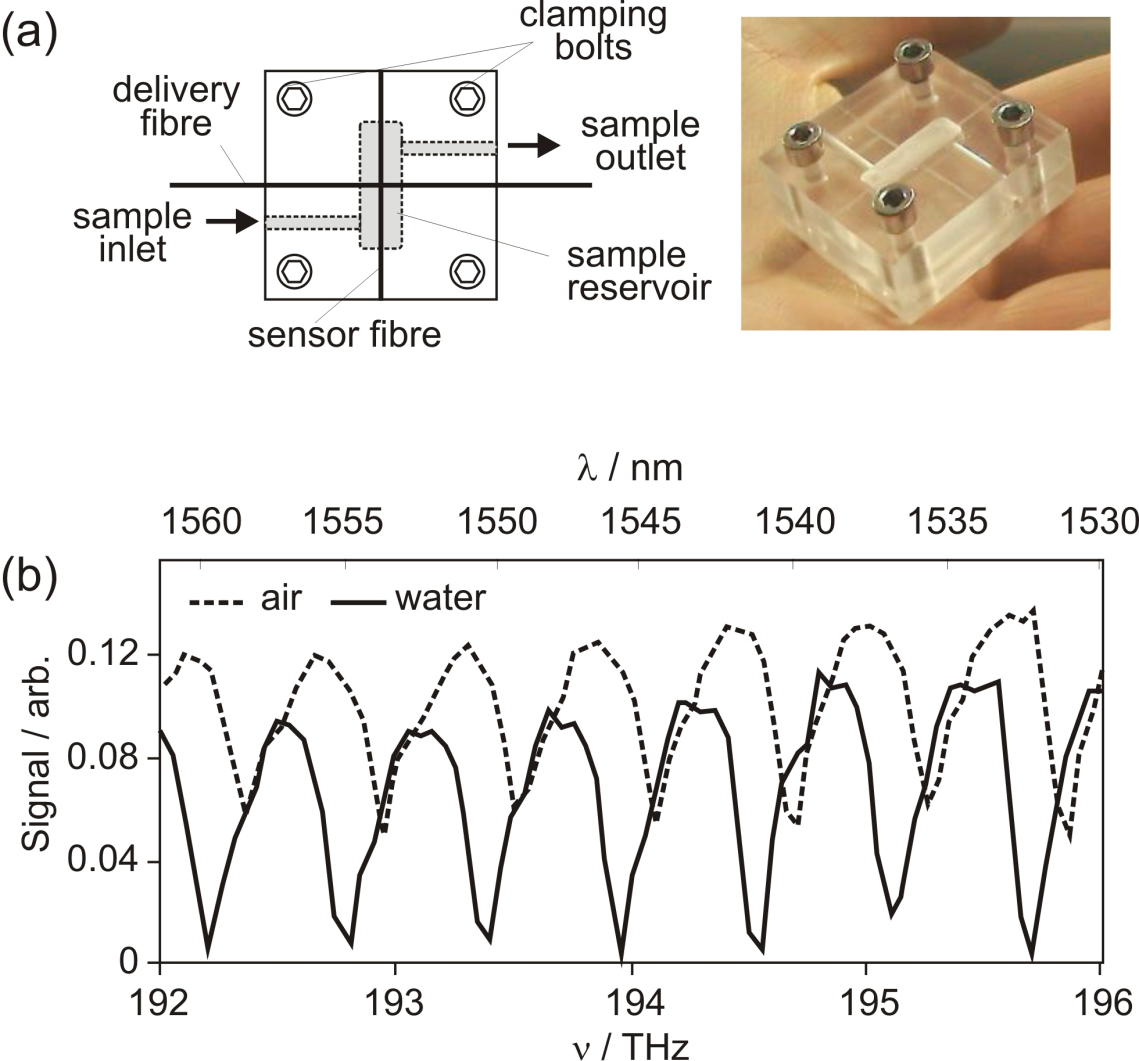
(a) Sample cell used for measurements in a liquid environment; (b) WGM spectra of a 125 *μ*m fibre measured in air and water.

Using the WGM spectroscopy technique for chemical sensing requires the ability to make measurements in aqueous solution. A small sample cell, shown in Figure 6(a), was designed for this purpose. The cell is fabricated from poly(methyl methacrylate) (PMMA) and consists of a reservoir to contain the sample of interest, with inlet and outlet ports to facilitate changes of sample. A pair of grooves crossing the reservoir at right angles serve to locate the delivery and sensor fibres within the cell.

#### Sensitivity to changes in bulk refractive index

Figure 6(b) illustrates the effect on the spectrum of a ‘bare’ resonator fibre when water is introduced to the reservoir. Changing the refractive index at the surface of the sensor fibre leads to a (reversible) shift in the whispering gallery mode resonance frequencies. However, there is little change in the modulation depth of the signal transmitted through the taper, implying that the coupling efficiency is similar in air and in water. The measured spectra are highly reproducible, indicating that the contact between the sensor and delivery fibre is not noticeably disturbed by water flow through the sample cell.

In order to determine the sensitivity of our fibre resonator to changes in the bulk refractive index, measurements were made in which ultrapure water (prepared using a Milli-Q Synthesis system) and ethanol were alternately flushed through the sample reservoir, with spectra recorded on each change of solvent. The associated wavelength shift averaged over all of the peaks in the spectrum, together with a one-standard-deviation error estimate, was 1.3 ± 0.1 nm. The refractive indices of water and ethanol at 1,550 nm and 293 K are [39] 1.323 and 1.354, giving a bulk sensitivity for our sensor of *S* = 42 ± 3 nm/RIU (RIU = refractive index units). A typical high resolution tuneable laser system has a bandwidth Δ*λ* of the order of 0.001 nm or better, which would yield a value of Δ*n* = Δ*λ/S* =∼ 2.4 × 10^−5^ for the detectable change in refractive index using our current setup.

Since in our experiments the electric field of the WGMs is relatively localised near the fibre surface, we can compare our calculated sensitivity with similar measurements made for capillary-based sensors. Our sensitivity compares favourably with the 16.1 nm/RIU reported by White *et al.* [34]. Zamora *et al.* showed that higher sensitivities could be achieved using capillaries with sub-micron wall thicknesses, and achieved sensitivities of 50–70 nm/RIU for TM modes, and 130–170 nm/RIU for TE modes in their resonators at refractive indices near that of water. In a related approach, Tan *et al.* [40] coated glass filaments with a high-refractive index surface guiding layer, and reported sensitivities of 50–70 nm/RIU, similar to that found in the present study.

#### Temperature Sensitivity

Changes in temperature have a small effect on both the diameter of the resonator (through thermal expansion) and the refractive indices of the resonator and the surrounding medium. For this reason, the temperature dependence of the WGM frequencies was evaluated prior to attempting any targeted binding studies in order to determine whether temperature-induced shifts in WGM frequencies should be considered as a source of error in our measurements.

Differences in the thermal expansion coefficients of the PMMA sample cell and the silica fibre meant that the cell could not be used reliably for these measurements. Instead, a simple alternative arrangement was used, in which the delivery and sensor fibres were fixed onto a glass substrate immersed in a water-filled reservoir resting on a hot plate. For each measurement, the hot plate was set to the required temperature, the system allowed to come to thermal equilibrium, and a spectrum recorded. The temperature was recorded using a digital thermometer.

Over the range from 20 to 40 °C, any shifts in WGM frequencies due to changes in temperature were essentially within our measurement uncertainty, and were at least a factor of ten smaller than the shifts observed in the analyte binding experiments described in the next section. This was in accord with the results of FDTD simulations in which the effects of the expected thermal variation of resonator diameter and refractive indices were explored. Temperature effects are therefore unlikely to have a significant impact on targeted sensing experiments carried out at a nominally constant temperature.

#### Targeted Detection of Unlabelled Streptavidin

Targeted sensing involves chemically modifying the surface of the fibre to enable specific binding of the analyte of interest. If the analyte is present in the sample solution then the change in refractive index at the fibre surface as the analyte binds to the fibre will lead to a shift in the WGM frequencies, providing a mechanism for detection. For example, using this technique, a sensor for a particular protein could in principle be fabricated by coating the fibre surface with the appropriate antibody. As outlined in the Introduction, a distinct advantage of WGM-based techniques over many existing biosensors is that they enable the detection of *unlabelled* molecular species.

Our proof-of-concept measurements utilised the biotin-streptavidin interaction. Biotin is a small B-complex vitamin molecule which binds through simple physisorption to bare silica surfaces, or may be bound covalently to suitably functionalised surfaces [41], while streptavidin is a 60,000 Dalton protein consisting of four 15,000 Dalton subunits, each containing one biotin binding site. The strong, highly specific and essentially irreversible binding interaction between biotin and streptavidin has made it a prototype system for modelling biological surface recognition processes. Ease of handling is a further advantage: both biotin and streptavidin are low-cost and stable in refrigerated buffer solution for several weeks. For the present experiments, a buffer solution of 0.2 mM BIS-TRIS (pH = 6.4) was used.

**Figure 7.**
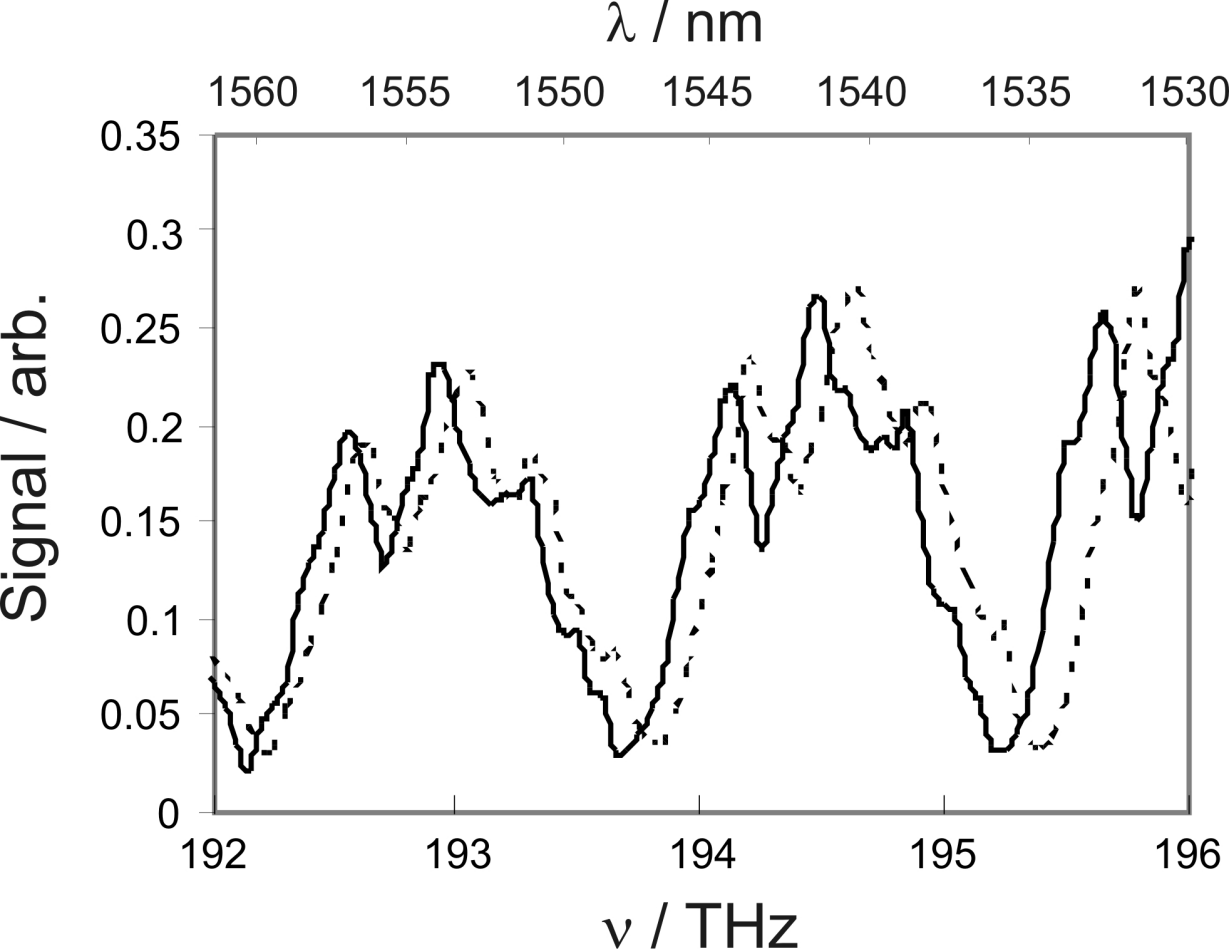
Frequency shift on binding of streptavidin to a biotinylated fibre. The spectrum of the biotinylated fibre is shown before and after the introduction of streptavidin to the sample cell as the dashed and solid lines, respectively.

A 45 *μ*m sensor fibre (fabricated from standard 125 *μ*m fibre using the same tapering rig employed to make the tapered delivery fibres) was employed in order to give well spaced WGMs within the frequency range of the laser. The sample cell containing the crossed delivery and sensor fibres was flushed first with ultrapure water and then with buffer solution. No shift in WGM frequencies was observed on switching between water and buffer, indicating that within our frequency resolution, the refractive index of the buffer may be assumed to be the same as that of water. After flushing with buffer solution, the fibres were treated with a 0.1 *μ*M solution of biotin (Sigma-Aldrich) in buffer and flushed again with pure buffer solution to ensure that only chemisorbed biotin remained attached to the fibre surface. In principle, we might expect to see a shift in WGM frequencies on binding of biotin to the fibre surface. However, no such shift was observed, indicating that our current frequency resolution is insufficient to detect the small refractive index changes associated with binding of a small molecule to the surface. After rinsing, a 1 *μ*M solution of streptavidin (Sigma-Aldrich) was admitted to the sample cell. Soon after injection of streptavidin into the sample cell, a shift of approximately 0.9 ± 0.2 nm in the WGM wavelengths was observed (see Figure 7). The shift did not increase when the streptavidin solution was left in the sample cell for longer times, and remained after rinsing of the sample cell with buffer solution. This indicates that it was caused by the refractive index change induced by streptavidin binding to the biotin-coated fibre surface rather than by streptavidin present in solution. In control experiments, no shift was detected when the resonator had not been pre-treated with biotin.

Equation 5 may be used to determine the surface density *σ*_p_ of detected streptavidin molecules. The excess polarisability of streptavidin is *α*_ex_ = 4*πɛ*_0_(3.2 × 10^−21^) cm^3^ [19], and the refractive indices of the resonator fibre and the solution at 1550 nm are 1.4629 and 1.323, respectively. Together with our value of 42 ± 3 nm/RIU for our bulk sensitivity and the observed fractional wavelength shift (averaged over all peaks) of *δλ/λ* ∼ (6±2) × 10^−4^, this yields a surface density of ∼ (2.7 ×0.9) × 10^17^ molecules m^−2^. We note that the evanescent field fraction in the 45 *μ*m fibre used to make the streptavidin measurements will be somewhat larger than for the 125 *μ*m fibre used to make our bulk sensitivity measurements, with FDTD simulations indicating a ratio of around 1.4 between the evanescent field fractions in the two fibres. A sensitivity increase of this order would yield a surface coverage of 1.9 × 10^17^ molecules m^−2^, still within our experimental errors.

A WGM in a 45 *μ*m resonator fibre excited with a 1 *μ*m taper corresponds to a surface area of approximately 1.4 × 10^−10^ m^2^ (we note that without detailed knowledge of the mode size within the resonator, we are unable to obtain a more accurate value for this parameter), so the frequency shift shown in Figure 9 is caused by adsorption of around 3.8 × 10^7^ molecules (6.3 × 10^−17^ moles).

Based on the somewhat limited information available on the dimensions of streptavidin, a monolayer would correspond to a surface density of ∼ 4 × 10^16^ m^−2^, somewhat lower than the result obtained above. This may simply be a reflection of the uncertainties in the values used for *α*_ex_ and the various refractive indices in the above calculation, or may indicate multilayer coverage of the fibre surface at the rather high concentrations used in our experiments.

Based on analysis of all of our existing experimental data on the biotin-streptavidin system, and taking into account the frequency resolution of the laser source, we estimate the detection limit for streptavidin using the current experimental setup to be around four times lower than the amount detected in the results reported above, *i.e.,* around 7 × 10^16^ molecules m^−2^.

While the streptavidin concentrations used in these experiments are higher than is likely to be found in biologically relevant systems, these experiments provide a first demonstration that targeted chemical sensing is possible using whispering gallery modes in optical fibres. Our detection sensitivity is currently limited primarily by the spectral resolution achievable with the fairly large minimum frequency step size of the communications laser employed to excite the WGMs. A second consideration is the reduction in Q factor resulting from the significant optical absorption of water at the wavelength of our laser system. Performing the experiments using D_2_O rather than H_2_O as the solvent could mitigate this issue, but since ideally a biological sensor will be optimised for operation in a range of aqueous environments, employing a tuneable laser source covering a different wavelength range is a more realistic long-term solution.

The FDTD simulations discussed earlier indicate that the ultimate resolution of the technique, dictated by the *Q* factor of the optical fibre resonator, is at least two to three orders of magnitude better than we are currently able to achieve. Alternative laser sources will be investigated in future work and should lead to a considerable improvement in our ability to detect small shifts in WGM frequencies. In addition, the observed shifts on binding of streptavidin vary according to the quality of the tapered delivery and sensor fibres used (irreversible binding of streptavidin to the fibre surface mean that new delivery and sensor fibres must be fabricated for each experimental run). A more robust process for creating reproducible tapers is needed in order to optimise this technique for chemical sensing. However, these early proof-of-concept results provide an encouraging indication that the fibre WGM technique could be developed into a robust and low-cost method for targeted sensing.

## 5. Conclusions

We have presented a whispering gallery mode sensor based on a a simple optical arrangement consisting of a pair of crossed optical fibres, and demonstrated potential applications in fibre size profiling and sensing of unlabelled chemical and biochemical species. A light source upgrade should allow us to improve the frequency resolution of the instrument to the point where detection of species at typical biological concentrations will be possible.
